# Prevalence of maternal smoking and environmental tobacco smoke exposure during pregnancy and impact on birth weight: retrospective study using Millennium Cohort

**DOI:** 10.1186/1471-2458-7-81

**Published:** 2007-05-16

**Authors:** Corinne Ward, Sarah Lewis, Tim Coleman

**Affiliations:** 1 Division of Epidemiology and Public Health, University of Nottingham, Nottingham, UK; 2 Division of Primary Care, University of Nottingham, Nottingham, UK

## Abstract

**Background:**

Meta-analyses of studies investigating the impact of maternal environmental tobacco smoke (ETS) on birth weight have not produced robust findings. Although, ante natal ETS exposure probably reduces infant's birth weights, the scale of this exposure remains unknown. We conducted a large, cohort study to assess the impact of ETS exposure on birth weight whilst adjusting for the many factors known to influence this.

**Method:**

Retrospective study using interview data from parents of 18,297 children born in 2000/2001 and living in the UK 9 months afterwards (the Millennium Cohort Survey). Comparison of birth weight, sex and gestational age specific (SGA) z score, birth before 37 weeks and birth weight < 2.5 Kg (LBW) in infants born to women exposed to: i) no tobacco smoke, ii) ETS only and iii) maternal smoking whilst pregnant.

**Results:**

13% of UK infants were exposed to ETS and 36% to maternal smoking ante natally. Compared to no ante natal tobacco smoke exposure, domestic ETS lowered infants' adjusted mean birth weights by 36 g (95% CI, 5 g to 67 g) and this effect showed a dose-response relationship. ETS exposure also caused non-significant increases in the adjusted risks of Low Birth Weight (<2.5 Kg) [OR 1.23 (95% CI, 0.96 to 1.58) and premature birth [OR 1.21 (95% CI, 0.96 to 1.51)], whilst the impacts of maternal smoking were greater and statistically significant.

**Conclusion:**

UK prevalences of domestic ETS exposure and maternal smoking in pregnancy remain high and ETS exposure lowers infants' birth weights.

## Background

Environmental tobacco smoke (ETS) exposure has a clinically significant, detrimental impact on public health [1,2] and so is an important issue for policy makers and clinicians. Maternal smoking during pregnancy impairs fetal growth [3-6]. and shortens gestation causing premature birth [7] with significant fetal and infant mortality and morbidity. ETS contains lower doses of the same toxins that smokers inhale [1], so maternal ETS exposure during pregnancy should have similar but less severe effects. If ETS exposure has even a small impact on fetal growth in the womb, this could translate into significant morbidity by increasing the numbers of high-morbidity, low birth weight (LBW) infants [8].

Studies investigating whether or not maternal ETS exposure during pregnancy affects birth outcomes have reported mixed findings [9,10]. A review [9] found significant heterogeneity between studies, but still presented synthesised findings and concluded that maternal ETS exposure during pregnancy reduced infants' adjusted mean birth weights by -24.0 g [95% CI -39.3 g to -8.6 g] and also increased the risks of babies being *either *"small for gestational age (SGA) or LBW at term" [9]. However, ETS exposure had no impact on the risk of either SGA or LBW at term alone and the reviewers noted that empirical studies were conducted in widely varied settings and often small and of poor quality, reporting only crude (i.e. non-adjusted) birth outcomes. Additionally, this review [9] searched only two databases for papers published before 1995, excluding those for which no English translation was available and it may not have determined the true impact of ETS on birth weight. Although a recent Surgeon General report concluded that ETS exposure reduces birth weight [11], this finding was based on the same review [9] without further literature searching or meta analyses. Consequently, previously calls for large, well-conducted cohort studies which have sufficient power to assess the impact of ETS exposure on birth outcomes whilst adjusting for the many factors known to influence these [9] remain relevant today[12]. We report the findings of such a study using data from the Millennium Survey, a contemporary UK epidemiological birth cohort, to investigate the UK prevalences of domestic ETS exposure and maternal smoking in pregnancy and the relative impact of these exposures on birth weight.

## Methods

This study utilised data collected by the UK Millennium Cohort Study (MCS), in which the parents of 18,819 children (18,553 single births), born over a 12 month period between 2000 and 2001, were interviewed when their children were 9 months old (72% response rate)[13]. Ethical approval was not needed for the analysis presented in this paper, because this involves secondary analysis of data from the MCS and interviewees gave consent for their anonymised, survey responses to be used for research purposes when they agreed to be interviewed for this [13]. Parents gave information regarding their social environment, socioeconomic status, smoking habits, obstetric history and birth outcomes. For the analysis presented here, we included only participants who provided information on their smoking status.

We obtained data from the UK Data Archive[14] and used STATA v.9[15] for analysis. We extracted self reported data on whether and how heavily mothers' had smoked at the start of pregnancy, and if and when, their habits had changed during this pregnancy. Similar data were also available for women's partners. These data were used to allocate cohort women to the following categories reflecting tobacco smoke exposure during pregnancy: i) no tobacco smoke exposure, ii) maternal active smoking at any point during pregnancy and iii) environmental tobacco smoke (ETS) exposure. ETS exposure was defined as occurring when a woman who did not smoke at all whilst pregnant had a partner who reported smoking during the pregnancy. This definition will underestimate total ETS exposure as, for example, occupational exposures were not assessed. We also derived variables with the following categories to indicate the amount smoked by women and their partners: non-smokers, smoking 1–10, 11–20 and over 20 cigarettes daily. Women without a partner and who did not smoke were defined as having no ETS exposure for analysis.

Birth weight data were obtained during interviews with parents (usually mothers) and 90% of recalled birth weights were validated from Parent Held Child Health Records (PHCHR) which the majority of the cohort used [16]. Gestational age at birth was derived from women's reported estimated date of delivery and their infants' birth dates (also validated from PHCHR). Finally, we extracted data on the following variables that are associated with birth weight[4] and/or shortened gestation [7] and had the potential to act as confounders: maternal age, body mass index at the start of pregnancy, ethnicity, educational level, income, gestational diabetes, alcohol intake and parity (number of previous live births).

### Analysis

The MCS survey participants were sampled by electoral ward, and using a weighted sampling strategy to ensure that that the sample was representative of all four UK countries and achieved higher than average numbers of respondents from economically-deprived areas and ethnic minorities. The MCS survey is thus a disproportionately stratified and clustered sample, and we used STATA survey to allow for clustering and for the weighted sampling to produce unbiased estimates of effect.

We calculated sex and gestational age specific (SGA) z scores from birth weight and gestation data using British birth weight reference data from 1990 and an algorithm, following a standard procedure [17,18]. SGA Z scores (or Standard Deviation Scores, SDS) are a measure of birth weight at a stated gestational age, and measured in standard deviations from the mean. Since this measure of weight is adjusted for gestation at birth, it is also a measure of fetal growth in utero. Birth weights under 2.5 kg were categorised as low birth weight (LBW) and gestation periods of less than 259 days were considered premature.

We used multiple linear regression to investigate differences between tobacco exposure groups in continuous outcomes (birth weight and SGA z scores) and logistic regression for dichotomous ones (LBW and prematurity). We examined the effects of potential confounders including maternal age, ethnic group, household income and maternal education, maternal body mass index at the outset of pregnancy, gestational diabetes, parity and maternal alcohol consumption during pregnancy, adjusting for all those that altered the size of effect of smoke exposure on any of the outcome variables. Missing values on each confounder were included as a category in the multivariate analysis, but we repeated the analysis including only those with complete data on all variables and confirmed that the results were very similar.

## Results

Of the 18,819 children in the Millennium cohort, 30 were triplets and 492 were twins. Consequently, of the 18,553 families within the study, 18,297 involved single births and these are those which contribute data to this analysis. There was complete smoking data at the start of pregnancy for 18,220 (99.6%) mothers, of whom 35% were smokers and about two-thirds of these were still continuing to smoke 6 months into their pregnancy. There was complete smoking data for 12,751 (85.5%) of their 14,859 partners, of whom 39% were smoking at the start of pregnancy, and most (94%) continued to smoke at 6 months of pregnancy. We were able to derive tobacco smoke exposure at the start of pregnancy for 16,756 (91.6%) of the mother-singleton infant pairs. Of these, 8100 (48.3%) mothers were not exposed to any tobacco smoke, 6397 (38.2%) were active smokers and 2259 (13.5%) were ETS exposed. After allowing for the sampling weights to attain figures representative of the UK population, the prevalence of exposure to maternal smoking and to ETS exposure at the start of pregnancy, were 36% and 13% respectively.

Table 1 shows the characteristics of women and babies included in the analysis, and those who were excluded due to missing smoking data. Those who were excluded were primarily non-smoking mothers with partners who did not provide smoking information and were more likely to be Asian, but otherwise similar in characteristics to the remaining non-smoking mothers. The distributions of maternal age, BMI, parity, household income, ethnicity, and maternal education differed between tobacco smoke exposure groups, and were treated as potential confounders in the analysis. Gestational diabetes was uncommon at 2% in non-exposed, 2.5% in ETS exposed, and 1.4% in active smokers.

**Table 1:** Maternal characteristics by smoking status (unadjusted for sample weighting)

**Maternal Characteristics**	***Non-smoker, non ETS exposed (n = 8100)***	***Non-smoker, ETS exposed (n = 2259)***	***Active smoker (n = 6397)***	***Excluded due to missing smoking data (n = 1541)***
**Maternal age at birth **Mean (SD)	29.6 (5.6)	28.7 (5.5)	26.2 (6.0)	29.2 (5.8)
**BMI **Mean (SD)	24.0 (4.4)	24.0 (4.8)	23.2 (4.4)	23.8 (4.4)
**Parity (previous live births) **N (%) *				
None	3310 (42.2)	862 (40.1)	2838 (45.2)	496 (36.0)
1–2	3952 (50.4)	1083 (50.4)	2882 (45.9)	750 (54.5)
3 or more	572 (7.3)	203 (9.4)	553 (8.8)	131 (9.5)
**Alcohol use in pregnancy **N (%)	622 (7.7)	157 (7.0)	684 (10.7)	98 (6.6)
**Ethnicity **N (column %) *				
White	6501 (80.4)	1701 (75.4)	6058 (94.9)	1018 (68.2)
Asian	872 (10.8)	412 (18.3)	76 (1.2)	364 (24.4)
Black	438 (5.4)	43 (1.9)	117 (1.8)	64 (4.3)
Mixed or other	275 (3.4)	100 (4.4)	134 (2.1)	46 (3.1)
**Education **N (column %) *				
Degree	1942 (24.0)	320 (14.2)	351 (5.5)	247 (16.4)
Diploma or A level	1714 (21.2)	440 (19.5)	789 (12.4)	278 (18.5)
O levels	3090 (38.2)	938 (41.6)	3401 (53.3)	633 (42.1)
None of the above	1342 (16.6)	558 (24.7)	1840 (28.8)	347 (23.1)
Income (£) N (column %) *				
**Less than 10,400**	1482 (19.9)	424 (20.3)	2514 (42.2)	256 (20.9)
**10,400 to 20,800**	2194 (29.5)	835 (40.0)	2058 (34.5)	453 (37.0)
**20,800 to 31,300**	1723 (23.2)	469 (22.5)	822 (13.8)	240 (19.6)
**Above 31,200**	2035 (27.4)	358 (17.2)	568 (9.5)	277 (22.6)

* where numbers in column do not add up to column total, this is due to missing data

After allowing for the sampling weights, the crude mean birth weights in non-exposed, ETS-only exposed and maternal smoking groups were 3.448, 3.389 and 3.279 Kg respectively (Table 2). Crude mean birth weight was lower in infants born to women in both tobacco smoke exposure groups than in non-exposed [for ETS exposed, mean difference = -59 g (95% CI -0.090 to -0.027) and for maternal smoking = -168 g (95% CI -0.191 to -0.146)]. Adjustment for maternal age, BMI, parity, alcohol use in pregnancy, household income, maternal education, maternal or the child's ethnic group and gestational diabetes, reduced the sizes of these effects to -36 g and -146 g respectively, but birth weight in both tobacco exposure groups remained significantly lower than in non-exposed. Table 2 shows a similar pattern of results for SGA z scores, though the reduction in the ETS exposed group compared to the non-exposed was not significant after adjustment for potential confounders.

**Table 2:** Associations between birth weight, SGA z score, and tobacco smoke exposure

	**Non-smoker, non ETS exposed**	**Non-smoker ETS exposed**	**Active smoker**
**Birth weight (in Kg)**	N = 8091	N = 2256	N = 6395
Mean Birthweight (SE)†	3.448 (0.007)	3.389 (0.014)	3.279 (0.009)
Crude mean difference compared to Non-Smoker, Non ETS exposed (95% CI)	-	-0.059 (-0.090 to -0.027) p < 0.001	-0.168 (-0.191 to -0.146) p < 0.001
Adjusted Mean difference* (95% CI)	-	-0.036 (-0.067 to -0.005) p = 0.025	-0.146 (-0.171 to -0.122) p < 0.001
**SGA z score**	N = 8024	N = 2228	N = 6338
Mean Z score (SD)†	0.077 (0.014)	0.006 (0.026)	-0.237 (0.016)
Crude mean difference compared to Non-Smoker, Non ETS exposed (95% CI)	-	-0.070 (-0.128 to -0.013) p = 0.016	-0.314 (-0.354 to -0.273) p < 0.001
Adjusted Mean difference* (95% CI)		-0.038 (-0.093 to 0.018) p = 0.18	-0.275 (-0.319 to -0.231) p < 0.001

* Adjustment was made for maternal age, BMI, parity, alcohol use, maternal education, ethnicity, income, gestational diabetes.

† All results are adjusted for the sampling design

Table 3 compares the incidence of low birth weight (LBW) and premature babies in the different groups. Maternal smoking significantly increased the risk of LBW (adjusted OR = 1.92 (1.60–2.29), p < 0.001) and ETS exposure was associated with a smaller and non-significant rise in LBW incidence (adjusted OR = 1.23 (0.96, 1.58), p = 0.1). Prematurity increased significantly with maternal smoking (adjusted OR = 1.25 (1.05–1.48), p = 0.013) but the smaller increase in the ETS-exposed group was not significant after adjusting for potential confounders (OR = 1.21 (0.96–1.51), p = 0.11).

**Table 3:** Associations between low birth weight, prematurity, and tobacco exposure

	**Non-smoker, non ETS exposed**	**Non-smoker ETS exposed**	**Active smoker**
**Low birth weight**	N = 8091	N = 2256	N = 6395
Birth weight < 2.5 Kg % †	4.4	6.0	8.3
Crude Odds Ratio compared to Non-Smoker, Non ETS exposed (95% CI)	1	1.39 (1.10, 1.77) p = 0.007	1.97 (1.68, 2.32) p < 0.001
Adjusted Odds Ratio * (95% CI)	1	1.23 (0.96, 1.58) p = 0.10	1.92 (1.60,2.29) p < 0.001
**Prematurity**	N = 8032	N = 2230	N = 6339
Gestation < 37 weeks % †	6.1	7.5	8.1
Crude Odds Ratio compared to Non-Smoker, Non ETS exposed (95% CI)	1	1.26 (1.01,1.57) p = 0.04	1.36 (1.17, 1.58) p < 0.001
Adjusted Odds Ratio * (95% CI)	1	1.21 (0.96, 1.51) p = 0.11	1.25 (1.05, 1.48) p = 0.013

* Adjustment was made for Maternal Age, BMI, parity, alcohol use, maternal education, income, ethnicity, gestational diabetes

† All results are adjusted for the sampling design

Table 4 shows how birth weight varies with the reported amount smoked by maternal smokers and with different levels of ETS exposure (i.e. number of cigarettes per day smoked by partners of non-smokers). In the adjusted model, there was a significant linear trend for reduced birth weight with increasing level of exposure for both maternal smoking and ETS exposures.

**Table 4:** Association between level of tobacco smoke exposure during pregnancy and birth weight (Kg)

	***Mean birth weight (SE)***	***Mean difference from non-smoker (95% CI)***	***Adjusted mean difference from non-smoker (95% CI)***	***P value for trend***
**ETS exposure (Level of partner cigarette consumption in non-smokers)**				
Non-smoker or no partner	3.448 (0.007)	-	-	
Smoker 1–10 cigs/day	3.386 (0.020)	-0.062 (-0.103, -0.021)	-0.027 (-0.067, 0.014)	0.007
Smoker 11–20 cigs/day	3.390 (0.024)	-0.058 (-0.107, -0.008)	-0.053 (-0.101, -0.004)	
Smoker 20+ cigs/day	3.407 (0.042)	-0.040 (-0.124, 0.044)	-0.059 (-0.141, 0.023)	
**Active Smoker (Level of Maternal cigarette consumption)**				
Non-smoker	3.429 (0.006)	-	-	
Smoker 1–10 cigs/day	3.325 (0.012)	-0.104 (-0.130, -0.078)	-0.086 (-0.114, -0.059)	< 0.001
Smoker 11–20 cigs/day	3.239 (0.014)	-0.190 (-0.220, -0.160)	-0.190 (-0.221, -0.159)	
Smoker 20+ cigs/day	3.152 (0.032)	-0.277 (-0.342, -0.213)	-0.275 (-0.341, -0.209)	

## Discussion

We found that tobacco smoke exposure in utero, remains a public health challenge for the UK as 13% of UK infants born between 2000 and 2001 were exposed to ETS and 36% to maternal smoking. Compared to no tobacco smoke exposure during pregnancy, domestic ETS exposure and maternal smoking significantly lowered infants' adjusted mean birth weights by 36 g (95% CI, 5 g to 67 g) and 146 g (122 g to 171 g) respectively and there was an exposure-response relationship between both reported exposures and adjusted mean birth weights. Maternal smoking caused significant reductions in adjusted SGA z scores, and the adjusted risks of LBW and premature births and ETS exposure also produced adverse changes in these outcomes, which were non-significant after adjustment for potential confounding.

This large cohort study has enough power to assess the impact of ETS exposure on birth outcomes whilst adjusting for the principal factors which affect these. Whilst some earlier studies were of a similar size, these reported only unadjusted changes in birth outcomes, and consequently may have over-estimated the impact of ETS [19,20]. Another strength of this study is that the validity of birth outcome data from the MCS is likely to be high. Data were collected by trained interviewers using a standardised interview schedule who were able to refer to parent held child health records in the vast majority of cases [16] and these records included birth weight and gestational age at birth.

A weakness of the study is the potential for recall bias in the retrospectively-obtained, self-reported data on tobacco smoke exposures. However, during pregnancy most women are very conscious of the need to restrict exposure of their developing babies to harmful substances like tobacco smoke, so most MCS participants will probably have been able to recall exposures correctly. Some might have deliberately concealed tobacco smoke exposure during pregnancy in order to give a socially desirable response to interviewers. If present, this bias would tend to reduce the observed effects of tobacco smoke on birth outcomes making it harder to detect differences. We did not have data on ETS exposure from other sources (e.g. recreational or occupational) and if any of these exposures were correlated with domestic exposure, then we may have over-estimated the impact of domestic ETS exposure alone. In contrast, around 10% of Millennium Cohort Survey households were shared by 'non-partner' adults (e.g. grandparents) and we know nothing about such adults smoking behaviour. Consequently, we may have under-estimated the prevalence of domestic exposure to ETS and a small number of infants may have been misclassified as not ETS-exposed when they actually were which would tend to weaken any apparent effect of ETS on birth weight. The fact that we demonstrated an exposure-response relationship between adjusted birth weight and reported domestic ETS exposure, however, provides evidence that this is a true effect and is consistent with studies which have found dose-response relationships between bio-chemical measures of tobacco exposures and birth weight [21-23].

Of infants born within the UK around the millennium, over one third had mothers who smoked, despite the widely publicised adverse impact of smoking on fetal development. In the US, however, only 10.2% of mothers smoked whilst pregnant in 2004, [24] which illustrates that the threat to public health from maternal smoking in pregnancy can be reduced and emphasises the need for action against this in the UK. For our analyses, we used tobacco smoke exposures which participants recalled from the time that their pregnancies were confirmed (i.e. the start of pregnancy) and also subsequent reports of changes in smoking status, so that active maternal smoking at any point during pregnancy could be determined. Only around 4% of women's partners changed their smoking status as pregnancies progressed, so the impact of ETS exposure was relatively constant during pregnancy and we observed a similar effect of ETS exposure on birth weight, irrespective of the point in gestation at which this was assessed. Maternal smoking was more likely to change during pregnancy than partner smoking, with 27% of mothers who smoked at the start of pregnancy reported to have stopped as their pregnancy progressed. Consequently, our findings may under estimate, to some degree, the impact of maternal smoking on birth outcomes, but we chose not to present these findings in detail because the focus of this manuscript is on the overall impact of ETS exposure on birth weight. We found that domestic ETS exposure lowered MCS infants' birth weights by approximately 25% of the reduction that one would expect from maternal smoking. Trends within the data suggest that domestic ETS exposure may also have caused some preventable morbidity and mortality by increasing the incidence of LBW infants and prematurity, but our sample size was not large enough for these effects to reach statistical significance. However, as the Millenium Cohort Survey contained no information on previous preterm birth, we have not been able to adjust for this important predictor of prematurity in subsequent births, so study findings relating to this outcome must be treated with some caution. Generally, though, higher levels of maternal ETS exposure appear to cause more harm to unborn children, so infants born to women exposed to the very highest ETS levels, such as bar staff, may be particularly at risk.

## Conclusion

Study findings emphasise the continued need for action against maternal tobacco smoke exposure in the UK in order to eliminate fetal harm that still results from this. They also provide further, strong evidence for an end to tobacco smoking in all enclosed areas. Some countries have introduced, or are considering, legislation to prevent smoking in enclosed public spaces [1] and, where introduced, this should have a positive effect on birth outcomes. As ETS exposure from domestic sources has a significant impact on birth weight, any similar legislation introduced into the UK should be accompanied by educational programmes to emphasise the fetal harm that may occur as a consequence of both active and passive maternal smoking within of pregnant women's homes.

## Competing interests

The author(s) declare that they have no competing interests.

## Authors' contributions

All authors participated in the design of the study. SL and TC had the idea for the study and developed a study protocol. CW and SL analysed data and all authors interpreted findings and wrote the paper. SL and TC are guarantors for data contained within the paper. All authors have read and approved the final version of this manuscript.

## Pre-publication history

The pre-publication history for this paper can be accessed here:


